# Thomas Hutchins

**Published:** 1849-01

**Authors:** 


Died at Glasgow, North Britain, on the 6th of February, THOMAS HUTCHINS, Esq., Surgeon Dentist, formerly of the city of Edinburg, Scotland.

